# Associations between body temperature and feed efficiency traits in lactating Holstein cows

**DOI:** 10.3168/jdsc.2024-0701

**Published:** 2024-12-12

**Authors:** Ligia Cavani, Larissa C. Novo, Faith S. Reyes, Bárbara M. Nascimento, Michael J. VandeHaar, Robert J. Tempelman, Kristen L. Parker Gaddis, Ransom L. Baldwin, José E.P. Santos, James E. Koltes, Heather M. White, Kent A. Weigel, Francisco Peñagaricano

**Affiliations:** 1 Department of Animal and Dairy Sciences, University of Wisconsin–Madison, Madison, WI 53706; 2 Angus Genetics Incorporated, Saint Joseph, MO 64506; 3 Form-A-Feed Inc., Stewart, MN 55385; 4 Department of Animal Science, Michigan State University, East Lansing, MI 48824; 5 Council on Dairy Cattle Breeding, Bowie, MD 48824; 6 Animal Genomics and Improvement Laboratory, Agricultural Research Service, USDA, Beltsville, MD 20705; 7 Department of Animal Sciences, University of Florida, Gainesville, FL 32608; 8 Department of Animal Science, Iowa State University, Ames, IA 50011

## Abstract

Differences in feed efficiency among dairy cows could be partially explained by changes in body temperature due to the heat increment. This heat represents chemical energy lost, and theoretically, it should be lower in more efficient cows. The aim of this study was to investigate the associations between 3 body temperature traits, namely average body temperature, consistency of body temperature, and change in body temperature after the largest meal of the day, with 4 feed efficiency traits: DMI, milk energy, metabolic BW, and residual feed intake (RFI) in lactating Holstein cows. Data were collected from 1,068 mid-lactation Holstein cows enrolled in 36 feed efficiency trials from 2020 to 2023 at 5 research stations across the United States. Temperature records were obtained using an automatic temperature logger placed vaginally for 2 wk. Average body temperature was calculated as the individual mean, daily and over the 2-wk period, and consistency of body temperature was calculated as the log-transformed variance of the deviations of individual records from the cow's mean. Change in body temperature was calculated for a subset of cows that used the roughage intake control system (n = 278) and was defined as the difference in temperature after and before the largest meal of the day. Data for DMI, milk energy, metabolic BW, and RFI were collected from the same cows for 6 to 7 wk during the feeding trials. The associations between body temperature traits and feed efficiency traits were assessed using univariate linear regression models including cohort (trial-treatment), temperature-humidity index (THI), and the interaction between THI and research station as independent variables. Partial correlations between body temperature and feed efficiency traits controlling for cohort were calculated. Additionally, the importance of body temperature traits on RFI calculation was also evaluated. Increased body temperature was associated with cow that ate less and were lighter. Consistency of body temperature was negatively associated with DMI and milk energy, showing that greater variance in body temperature is linked to higher DMI and more milk energy. Smaller changes in body temperature after the largest meal were associated with lower DMI and milk energy. Adding body temperature traits to the RFI model did not change the coefficient of determination (adjusted R^2^), which remained ∼0.86. We found that cows that eat less have higher body temperatures, with less fluctuation throughout the day, compared with cows that consume more feed and produce more milk. Overall, our results suggest that vaginal temperature did not capture the heat increment expected in high RFI cows.

## Highlights

• Higher body temperature was associated with lower dry matter intake (DMI) and metabolic body weight.
• Greater variation in body temperature was associated with greater DMI and milk energy.
• Smaller post-meal body temperature changes were associated with lower DMI and milk.

## Graphical Abstract

**Figure:**
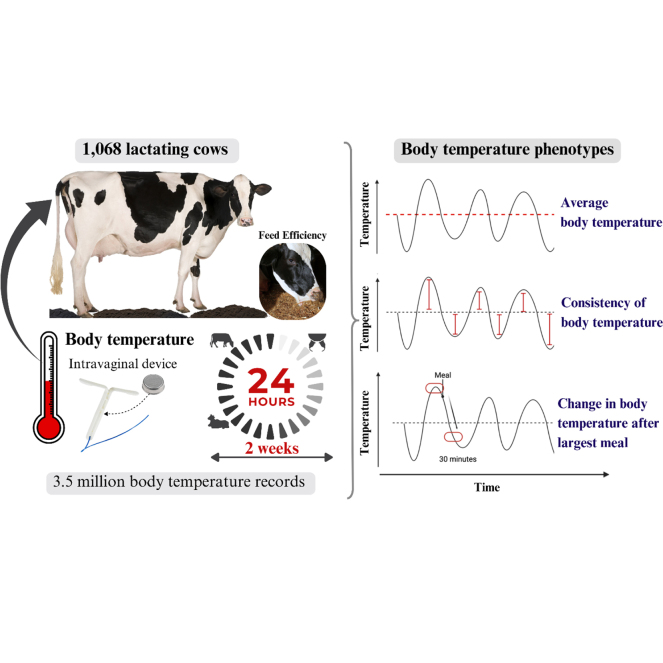
**Summary:** Differences in energy expenditure could be related to changes in body temperature in lactating dairy cows. Here, body temperature was collected on 1,068 mid-lactation Holstein cows using an automatic temperature logger placed vaginally for 2 weeks, totaling 3.5 million body temperature records. We developed 3 phenotypes for body temperature: average body temperature, consistency of body temperature, and change in body temperature after the largest meal. Average body temperature was calculated as the individual mean, daily and over the 2-week period; consistency of body temperature was calculated as the log-transformed variance of the deviations of individual records from the cow's mean temperature; and change in body temperature was calculated as the difference in temperature after and before the largest meal of the day. Overall, our results suggest that vaginal temperature did not capture the heat increment expected in high residual feed intake cows.

Thermoregulation is an important aspect of metabolism and might play a key role in the biological mechanisms involved in economically important traits in dairy cows. Not surprisingly, body temperature has been used for the early detection of diseases (Smith and Risco, 2005; Adams et al., 2013), prediction of reproductive events (Burfeind et al., 2011; Suthar et al., 2011), and evaluation of the impact of heat stress (Scanavez et al., 2017; Lees et al., 2019). In addition, differences in feed efficiency among animals could be partially explained by changes in body temperature. Indeed, the rationale behind the hypothesis of body temperature being associated with feed efficiency, regardless of heat stress impact, is that the heat increment of feeding is due to metabolism, digestion, and product formation (Herd and Arthur, 2009). This heat represents chemical energy lost during the digestion of feed, metabolism of nutrients, and product synthesis, and theoretically, it should be lower in more efficient cows. However, inconclusive results have been found regarding the relationship between body temperature and feed efficiency in lactating dairy cows (DiGiacomo et al., 2014; Fischer et al., 2018). One factor that could explain the challenge of unlocking this association is the fact that cows are homeotherms, so their regulation of body temperature involves a balance process between heat production and heat dissipation (McArthur and Clark, 1988; Silanikove, 2000).

The objective of this study was to investigate the association between body temperature and feed efficiency in lactating Holstein cows. The body temperature of each cow was assessed through high-frequency vaginal temperature recordings taken every 5 min for 2 wk. Daily and overall averages for body temperature, consistency of body temperature, and change in body temperature after the largest meal of the day were associated with DMI, milk energy, metabolic BW, and residual feed intake (**RFI**).

Data were collected from 1,088 mid-lactation Holstein cows in 36 trials between June 2020 and December 2023 at 5 research stations: the University of Wisconsin–Madison (Arlington, WI, and Marshfield, WI; 328 cows), Michigan State University (East Lansing, MI; 260 cows), University of Florida (Gainesville, FL; 229 cows), Iowa State University (Ames, IA; 212 cows), and the USDA-Agricultural Research Service Animal Genomics and Improvement Laboratory (Beltsville, MD; 59 cows). All procedures were approved by the corresponding Institutional Animal Care and Use Committees. Body temperature was collected with a Thermochron iButton device (Embedded Data Systems, Lawrenceburg, KY) that was placed in a blank intravaginal device (CIDR, Zoetis US, New York, NY). To avoid malfunction and recording failure, the CIDR with the temperature device was assembled following an internal protocol consisting of filling the gaps between the device and CIDR and covering it with shrink wrap and electrical tape to ensure it was waterproof. Cows in early stages of pregnancy or cows enrolled in insemination protocols were not included for temperature collection. Each eligible cow received one device, and temperature was recorded every 5 min for 2 wk on average, with a precision of 0.0625°C. Animals with signs of severe discomfort or lesions were removed from the study. Cows with fewer than 7 d of records were excluded, and individual temperature records outside 3 SD from the cow's mean vaginal temperature were not considered. The final dataset consisted of 1,068 lactating Holstein cows with a total of 3.5 million body temperature records, with an average of 12.5 ± 2.4 d of vaginal temperature records taken every 5 min per cow.

These trials were part of the routine data collection for the national genetic evaluation of feed efficiency in dairy cattle in the United States. Daily feed intakes were measured via roughage intake control system (Hokofarm Group, Marknesse, the Netherlands), Calan Broadbent Feeding System (American Calan, Northwood, NH), GrowSafe System (Vytelle, Calgary, AB, Canada), or manual weigh-back of refusals. In addition, milk yield, milk composition, and BW were recorded. Details of data collection protocols varied by station, but generally DMI and milk yield data were measured daily, milk components were analyzed at least once per week, and BW were measured daily, weekly, or on 3 consecutive days at the beginning, middle, and end of each trial (Tempelman et al., 2015; Cavani et al., 2023). Milk energy, in Mcal/d, was calculated weekly using the following equation (NASEM, 2021), and then averaged by cow to obtain the milk energy (**MilkE**) per individual:

MilkE = (0.0929 × fat % + 0.0585 × true protein % + 0.0395 × lactose %) × milk yield.

Similarly, BW were recorded manually at the beginning, middle, and end of the trial in most studies, whereas other studies recorded BW manually on a weekly basis, and others recorded BW daily using walk-over scales. Because the experiments lasted only a few weeks, we used linear regression of measured BW on day of trial to estimate missing BW records. Metabolic BW was calculated as the cow's average BW^0.75^. Residual feed intake for each cow was calculated using the following linear model:

DMI = DIM + Lact + Cohort + *b*_1_MilkE + *b*_2_mBW + *b*_3_ΔBW + *e*,

where DMI in the average DMI, DIM is the midpoint days in milk with 9 levels (grouped by 15-d periods, from 60 to 195 DIM), Lact is the lactation number with 4 levels (1, 2, 3, and 4+), Cohort is the trial-treatment with 72 levels, MilkE is the average secreted milk energy with partial regression coefficient *b*_1_, mBW is the average metabolic BW with partial regression coefficient *b*_2_, ΔBW is change in BW (difference of BW between the end and the beginning of the trial period) with partial regression coefficient *b*_3_, and *e* is the random residual of the model assumed to follow a normal distribution $e∼\left(0,I\sigma_{e}^{2}\right).$ This residual represents the RFI value for each cow.

The following body temperature traits were considered in this study: average body temperature per day and during the trial period, consistency of body temperature per day and during the trial period, and average change in body temperature after the largest meal of the day. Consistency of body temperature was calculated as log(var × 100), where log is the natural logarithm and var is the variance of the deviations of individual records from the cow's mean. Cows with smaller values of log(var × 100) indicate less body temperature variation, and hence more consistency. Change in body temperature after the largest meal was calculated as the body temperature 20 min after the end of the meal minus the body temperature at the beginning of the meal. The beginning and end body temperatures were considered as the average of 3 consecutive temperature measurements. The largest meal of the day consisted of the meal with the highest intake. The meal criterion used was 26.4 min, which means that any pair of feeder visits separated by less than 26.4 min were considered part of the same meal. Meal criterion was determined by fitting a mixture model of the log_10_ frequency distribution of the interval between feeder visits using maximum likelihood (Brown et al., 2022; Cavani et al., 2022). Phenotypes for change in body temperature after the largest meal were available only for cows at the University of Wisconsin–Madison Emmons Blaine Dairy Cattle Research Center (Arlington, WI; 278 cows), where there is a roughage intake control system (RIC, Hokofarm Group) that allows the identification of the largest meal per day. Descriptive statistics for all body temperature and feed efficiency traits are shown in Table 1. Note that the feed efficiency data included more cows than those having temperature data because RFI was calculated considering all cows enrolled in the same trials in which vaginal temperature records were collected.

**Table 1:** Descriptive statistics for body temperature traits and feed efficiency traits in lactating Holstein cows

Trait	Cows (n)	Mean	SD	Minimum	Maximum
Body temperature					
Average body temperature (°C)	1,068	38.7	0.11	38.3	39.0
Average body temperature per day (°C)	1,068	38.7	0.14	37.9	39.2
Log-variance body temperature	1,068	1.46	0.38	0.27	2.65
Log-variance body temperature per day	1,068	1.29	0.50	−1.50	2.98
Change in body temperature after largest meal^1^ (°C)	278	−0.27	0.12	−0.65	0.09
Feed efficiency					
DMI (kg/d)	1,542	26.7	4.22	13.5	38.6
Secreted milk energy (Mcal/d)	1,542	31.4	5.39	11.3	48.5
Metabolic body weight (kg)	1,542	131.7	12.5	97.7	171.2
RFI (kg)	1,542	0	1.49	−8.23	9.75

1 Calculated as the body temperature 20 min after the end of the meal minus the body temperature at the beginning of the meal.

The associations between body temperature traits and feed efficiency traits were assessed using linear regression models containing the body temperature phenotype and the cohort (trial-treatment) as independent variables. The temperature-humidity index (**THI**) during the period ± 3 d when body temperature was recorded was considered as a covariate in the models. Additionally, the interaction between THI and research station was considered in the model. For the daily body temperature traits, i.e., average body temperature per day and consistency of body temperature per day, their associations with daily DMI were assessed using linear mixed-effects models, considering the effects of cohort and daily THI as fixed effects, interaction between THI and research station, and cow as a random effect.

The THI was calculated as follows:

$$THI=\left(1.8\;\times \;t_{{\;}^{\circ }C}+32\right)-\left(0.55-0.0055rh_{\%}\right)\;\times \;\left(1.8\times t_{{\;}^{\circ }C}-26\right),$$

where t_°C_ is the mean air temperature in degrees Celsius at ∼2 m, and rh_%_ is the mean relative humidity as a percentage. Weather data were obtained using the nasapower package (Sparks, 2018, 2024) in R (version 4.2.2; https://cran.r-project.org/web/packages/nasapower/nasapower.pdf) considering latitude and longitude of the research station.

Partial correlations between body temperature and feed efficiency traits controlling for cohort were calculated. Additionally, to evaluate whether body temperature can explain part of the difference observed in feed efficiency, we compared the goodness-of-fit, calculated as adjusted R^2^, of the RFI models (described previously) with and without body temperature traits.

Table 2 shows the estimated regression coefficients (slopes) of feed efficiency traits on body temperature traits. Increased body temperature was associated with lower DMI and lower metabolic BW. Greater variation in body temperature (less consistency) was associated with greater DMI and MilkE. Interestingly, the change in body temperature after the largest meal was, on average, negative (−0.27; Table 1), showing that after food ingestion, vaginal temperature decreased. Therefore, the values in Table 2 for the change in body temperature trait indicated an association between an increase in vaginal temperature after a meal, relative to vaginal temperature before the meal, and lower DMI as well as smaller MilkE.

**Table 2:** Association between body temperature traits with DMI, milk energy (milkE), metabolic BW (mBW), and residual feed intake (RFI) in Holstein cows

Trait	DMI (kg/d)		milkE (Mcal/d)		mBW (kg)		RFI (kg)	
	Slope	*P*-value	Slope	*P*-value	Slope	*P*-value	Slope	*P*-value
Average for the trial period								
Body temperature (°C)	−2.55	0.009	−1.13	0.43	−15.92	<0.001	0.26	0.60
Log-variance body temperature	0.61	0.06	1.87	<0.001	−0.60	0.61	−0.07	0.70
Change in body temperature after largest meal^1^ (°C)	−5.00	0.009	−5.95	0.02	1.56	0.81	−2.12	0.01
Daily average								
Body temperature (°C)	−2.30	<0.001						
Log-variance body temperature	0.36	<0.001						

1 Calculated as the body temperature 20 min after the end of the meal minus the body temperature at the beginning of the meal.

Partial correlations are depicted in Figure 1. Partial correlations between change in body temperature after meal and DMI, MilkE, metabolic BW, and RFI were −0.06, −0.08, 0.01, and −0.08, respectively, and were not significant (*P* > 0.05). These results were not included in Figure 1 because the change in body temperature trait was calculated from a subset of cows.

**Figure 1:**
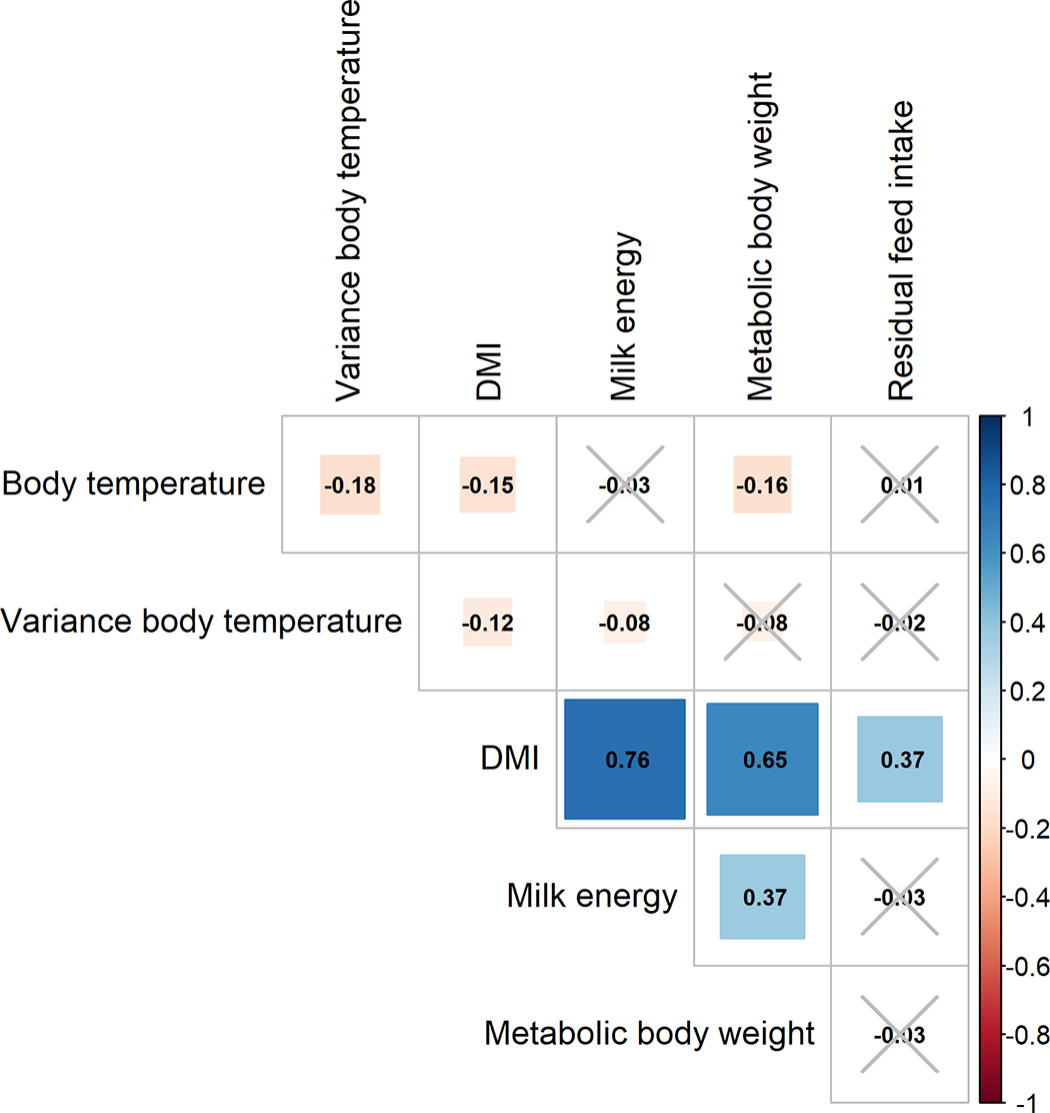
Partial correlations among body temperature traits and DMI, milk energy, metabolic BW, and residual feed intake (RFI) controlling for cohort in mid-lactation Holstein cows. Values marked with a cross were not significant (*P* > 0.05). All traits were averaged during the trial period. Variance in body temperature represents the consistency of body temperature, calculated as log(var × 100), where var is the variance of the deviations of individual records from the cow's mean.

Adding average body temperature or consistency of body temperature to the RFI model did not change the adjusted R^2^, which remained at 0.86. Similar results were observed when change in body temperature after a meal was added to the RFI model.

Despite not being useful as an important variable to incorporate into RFI models, we found some interesting associations between vaginal temperature and traits related to feed efficiency, such as DMI, MilkE, and metabolic BW. In cattle, the normal core body temperature is typically around 38.6 ± 0.5°C (Andersson and Jónasson, 1993). High-frequency measurements of rectal and vaginal temperature have been considered reliable methods to monitor core temperature in dairy cows (Suthar et al., 2013). Also, rumen temperature, when corrected for water intake, can reflect core body temperature even though it typically exhibits higher temperatures compared with core body temperature. (Suthar et al., 2013; Fischer et al., 2018; Godyń et al., 2019). The association between body temperature and feed efficiency could be related to the fact that less efficient cows may have a greater heat increment. However, a simple body temperature average may not be informative enough to capture such associations. Indeed, no significant association was found between average body temperature and RFI, which agrees with findings from other studies in lactating dairy cows (DiGiacomo et al., 2014; Fischer et al., 2018). Still, both the average body temperature over 2 wk and the daily body temperature were associated with lower DMI.

The large dataset used in this study, both in number of cows and records per cow, provides an opportunity to explore more body temperature traits beyond simple averages. The second phenotype calculated herein was log-transformed variance of the deviations from the cow's average temperature, which depicts consistency of an animal's body temperature. Cows with less consistent body temperatures (more variation), both daily and over the entire period, exhibited greater DMI. In addition, greater variation in body temperature was associated with higher MilkE. Going deeper, the third phenotype analyzed was the change in body temperature after the largest meal of the day. Interestingly, cows that showed a smaller change in body temperature after their largest meal, meaning their post-meal vaginal temperature was similar to or higher than their pre-meal temperature, exhibited lower DMI and lower MilkE.

Overall, the results presented in this work suggest that vaginal temperature did not reflect or capture the heat increment expected in high RFI cows. Indeed, the decrease in vaginal temperature after the largest meal may be explained by the redirection of blood flow from the extremities to the gastrointestinal tract, which is expected after food ingestion to prioritize digestion and nutrient absorption (Sangsritavong et al., 2002). Larger changes in body temperature were associated with greater DMI and MilkE, indicating that cows with a greater temperature decrease after feeding consumed more feed and allocated more energy toward milk production. Although vaginal temperature may not capture the heat increment, as a measure of body temperature, it was still associated with traits related to feed efficiency in lactating dairy cows. We found that cows that eat less have higher body temperatures, with less fluctuation throughout the day, compared with cows that consume more feed and produce more milk. However, it is well known that cows that eat more produce more heat (West et al., 2003). Therefore, vaginal temperature must not be a good indicator of the rate of heat production.
